# Study on the permeability characteristics of different geotextiles under weft and warp stretching

**DOI:** 10.1371/journal.pone.0306057

**Published:** 2024-06-27

**Authors:** Xiaolei Man, Jing Liu, Xueli Liu, Chengbin Lu, Yun Chen

**Affiliations:** 1 Geosynthetics Applied Research Centre, College of Civil and Architecture Engineering, Chuzhou University, Chuzhou, China; 2 College of Material and Chemical Engineering, Chuzhou University, Chuzhou, China; National Textile University, PAKISTAN

## Abstract

Geotextiles are excellent anti-filtration materials commonly used in the field of water conservancy engineering; however, the factors affecting the water permeability performance of geotextiles under stressed states during operation have not been fully identified. To investigate the effect of unidirectional stretching on the water permeability of geotextiles, a unidirectional rheological head infiltration test was conducted on the geotextiles using a self-developed test apparatus. In addition, the water permeability of geotextiles with different thicknesses and tensile states was calculated using a set of water permeability calculation methods based on the nonlaminar flow state of geotextiles. The results showed that the water permeability of the W120 geotextile samples initially decreased and then increased under warp stretching and gradually increased under weft stretching. However, the water permeability of the W200 geotextile samples initially decreased and then increased under both warp and weft stretching. Therefore, the thickness of the geotextile affected its permeability properties.

## 1. Introduction

In engineering fields such as water conservancy, electricity, mines, highways and railways, geotextiles are permeable geosynthetics [1] with high strength, corrosion resistance, and convenient construction and can form an inverted filter layer inside the soil to effectively prevent the erosion of soil materials, drain excess water and exhaust air, and decompose and transfer stress to protect the soil from being damaged by stress. With these advantages, geotextiles are widely used in the reinforcement and stabilization of railroad roadbeds, maintenance of pavements, protection of dams, segregation of construction materials, environmental protection, flood control, emergency response, and other projects [2–5].

Geotextiles have good isolation, filtration, and drainage functions in various engineering applications; therefore, a great deal of research has been conducted on their water permeability worldwide. Mukhopadhyay et al. reported the influence of different degrees of water pollution on the water permeability of nonwoven geotextiles and reported that the water permeability of geotextiles decreased with increasing pollutant concentration in water [6]. Pak et al. studied the drainage and filtration of geotextiles of different types and thicknesses under different hydraulic gradients and pressures and reported that these performances were closely related to the type, thickness, and porosity of geotextiles within a certain range of hydraulic gradients [7]. Moreover, relevant studies have shown that the drainage of geotextiles is also closely related to their water content. Although unsaturated geotextiles have good water permeability and drainage properties, their permeability is affected by the changes in the pore structure and water flow field [8]. On this basis, Mccartney et al. evaluated the permeability, porosity, and pore size distribution of unsaturated nonwoven geotextiles during application and operation through a series of hydraulic tests of geotextiles with different pore sizes and materials; they concluded that unsaturated nonwoven geotextiles had excellent hydraulic performance as effective hydraulic isolation materials for preventing soil erosion and protecting groundwater resources [9]. With the increasing strength requirements of geotextiles in practical engineering, multilayer geotextiles have gradually begun to be applied. Liu et al. studied the influence of the number of layers of geotextiles on the permeability through experiments and numerical simulation [10]. They found that the permeability of multilayer nonwoven geotextiles was better than that of single-layer nonwoven geotextiles. In addition, they found that the permeability of multilayer nonwoven geotextiles could be optimized by changing the number of layers and material properties; however, when the number of layers increased to a certain extent, the permeability gradually decreased. In addition, with the change in actual engineering diversity, the selection of geotextile materials has gradually attracted the industrial interest. Relevant research shows that different types of geosynthetics exhibit different performance characteristics in engineering, and appropriate materials need to be selected with respect to the specific engineering requirements [11, 12].

However, in engineering applications involving geotubes, geotubes are subjected to a stressed state due to the current shock during filling and stacking into dams and during use; therefore, the influence of geotextile deformation on water permeability need to be considered in future research. Palmeira E M et al. used a three-dimensional model simulation to explore the variation trend of the filtration opening size of a geotextile under different tension and constraint conditions [13]. They found that different tension and constraint conditions affected the filtration opening size of the geotextiles and changed their porosities and permeabilities. More subtle research showed that tensile strain the pore size and distribution of acupuncture nonwoven geotextiles, and affected the permeability [14]. In the field of geotube engineering, geotextiles are in direct contact with soil. When exploring the stress-strain relationship and deformation properties of geotextiles in soil, geotextiles in soil exhibited good ductility and deformation properties, and the latter property was affected by the soil [15]. To facilitate the research of geotubes, Liu Weichao studied the filling property and calculation theory by combining laboratory tests and numerical simulation [16]. The results showed that geotextile tubes had good filling properties and could effectively control the permeability and porosity of the soil, thus improving the consolidation and stability of the soil. Moreover, he proposed a calculation theory of geotube filling characteristics based on finite element analysis, which could accurately predict the filling effect of geotextile tubes under different conditions.

The above research shows that geotextiles have wide application prospects in engineering, and their permeability is one of the key factors in their application; therefore, further exploration and research on the permeability of geotextiles have important theoretical and practical significance. Man et al. suggested that the mechanical properties of geotextiles differed between warp and weft stretching, but they did not consider the influence of the thickness of geotextiles on their mechanical properties during warp and weft stretching [17]. Furthermore, different woven auxetic structures of fabrics significantly affect their tensile strength in the warp and weft directions [18]. Therefore, in this study, woven geotextiles with specifications of 120 g/m^2^ and 200 g/m^2^ (abbreviated below as W120 and W200) were selected to be stretched to strains of 0%, 3%, 6%, and 9% in the warp and weft directions, respectively; then, an independently developed intelligent multi-strain geotextile permeameter was used to carry out a variable water head permeability test to explore the variation trend of the water permeability of different geotextiles under uniaxial tension as a reference for subsequent researchers. Moreover, the theoretical calculation formula for laminar and nonlaminar flow was deduced; based on a comparison with test data, a more reasonable calculation formula was derived for geotextile permeability, and this calculation formula could be used as a reference for actual projects.

## 2. Laboratory tests

### 2.1. Apparatus

**Fig 1:**
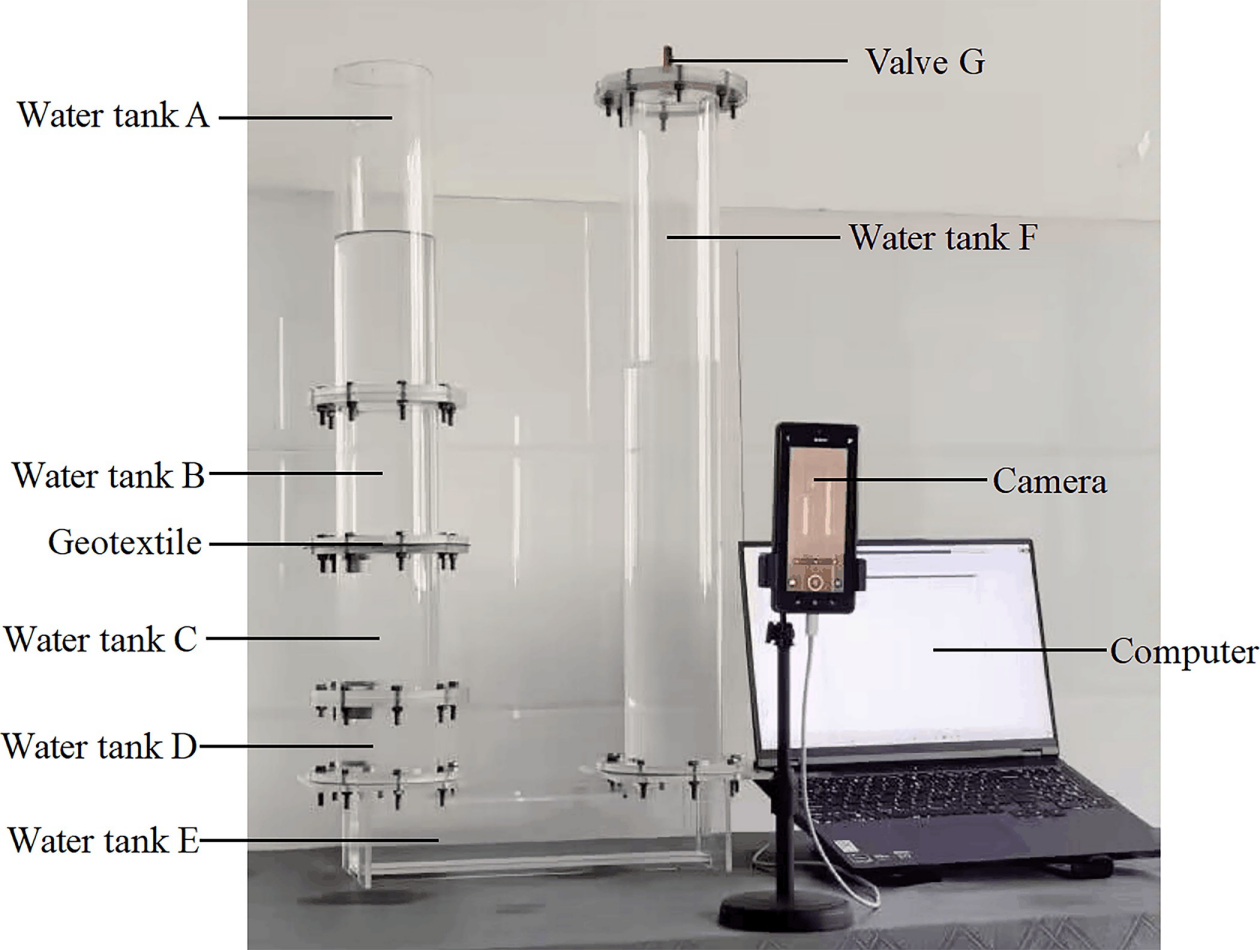
Intelligent multi-strain geotextile permeameter.

**Fig 2:**
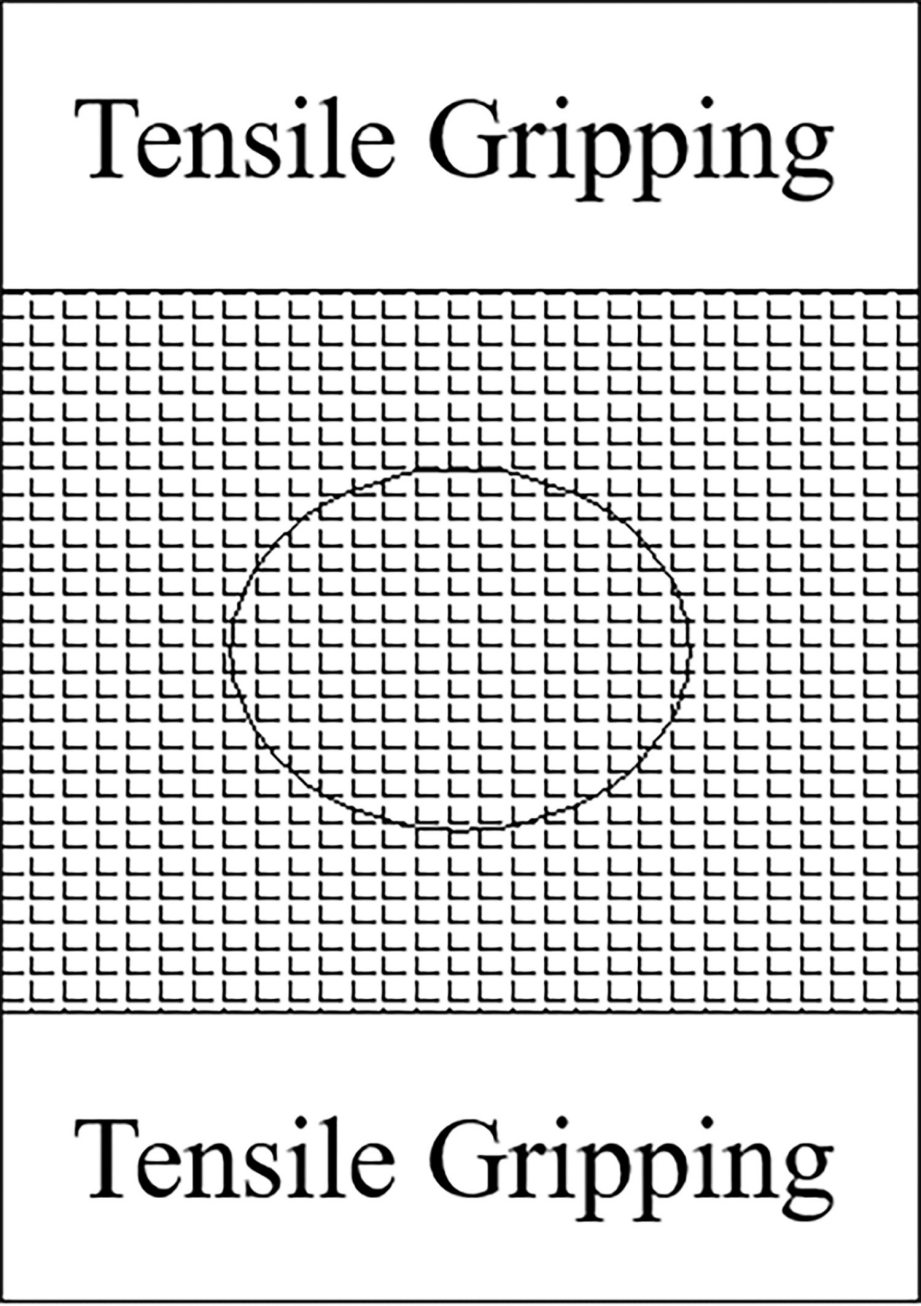
Schematic diagram of the test fabric sample.

The testing apparatus adopts the independently developed intelligent multi-strain geotextile permeameter, as shown in Fig 1. This apparatus consists of a variable water head permeameter system and a data acquisition and analysis system. The permeameter is made of organic glass, numbered from left to right as water tank A (inner diameter 95 mm, wall thickness 5 mm, height 340 mm, and lower connecting flanges), water tank B (inner diameter 95 mm, wall thickness 5 mm, height 150 mm, and connecting flanges at both ends), water tank C (inner diameter 95 mm, wall thickness 5 mm, height 150 mm, and connecting flanges at both ends), water tank D (inner diameter 95 mm, wall thickness 5 mm, height 80 mm, and connecting flanges at both ends), water tank E (inner diameter 70 mm, wall thickness 5 mm, height 98 mm, and connecting flanges at both ends), water tank F (inner diameter 95 mm, wall thickness 5 mm, height 720 mm, and connecting flanges at both ends), and valve G made of organic glass. The data acquisition and analysis system consisted of a camera and a computer. A soft ruler with an accuracy of 1 mm was fixed on the outer casing of tank F to measure the water level. In this study, two kinds of geotextiles W120 and W200 commonly used in geotube embankment engineering were selected as test samples. Fig 2 shows a schematic diagram of the fabric samples. During the test, A, B, C, D, E, and F were connected by flanges, and the flanges between B and C were sandwiched by woven geotextiles and fixed by bolts.

### 2.2. Testing procedure

First, the geotextile samples of W120 and W200 needed for the test were prepared and cut into circles or rectangles of appropriate sizes. The whole test was divided into the following five processes.

Infiltration: To eliminate impurities and air during sample clearance, the samples were soaked in deionized water for 12 hours.

Instrument assembly: The soaked geotextile samples were held on a high- and low-temperature tensile tester along the short edge direction and stretched in the warp and weft directions under the specified working conditions. Stretching in the direction of a geotextile’s warp yarns is defined as warp stretching, and stretching in the direction of a geotextile’s weft yarns is defined as weft stretching. Duct tape was used to hold the tensile fabric with flanges on test instruments B and C, and the flanges were clipped with an electric soldering iron, punched, and reinforced with bolts. Subsequently, B-geotextile-C was removed from the high- and low-temperature tensile tester. In the same way, the samples under various working conditions were connected to the instrument by flanges and bolts, and duct tape was added at the flange joint to ensure that the seams of all parts of the test device were sealed and that no water leakage occurred during the test.

Water injection: Water was slowly and steadily injected from the upper end of the water tank F after assembling the instrument. When the water level on the left side of the instrument was about to overlap with the fabric, a dropping bottle was used to ensure that the water and the fabric were in a state of contact, and the injection was stopped.

Water level calibration: The upper ends of G and F were connected with bolts through flanges, and the valve was close. The mixture was allowed to stand for 5–10 minutes to ensure that the right system was sealed and that the water level did not noticeably change. The water was slowly injected from the upper end of water tank A until the water level was flush. The mixture was allowed to stand for 5–10 minutes to ensure that the water level did not drop.

Test: A camera and computer were set up in a fixed position, and the camera was started; when the valve was opened at G, the test was started.

### 2.3. Data processing

**Fig 3:**
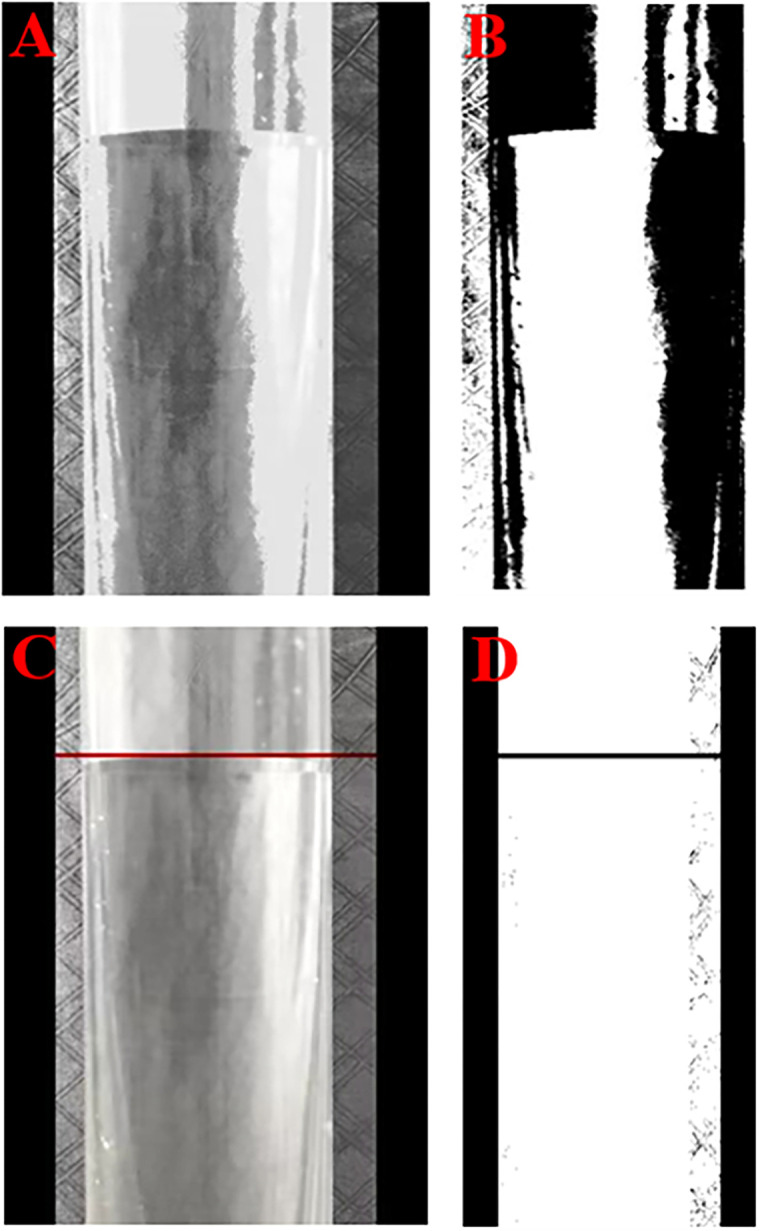
Program processing. (A) grayscale image, (B) binary image, (C) water level red line image, and (D) water level binary image.

The shot video was imported into an independent written program, and the variation parameters were logically analyzed. The result of the program processing is shown in Fig 3. The program was run as follows:

First, the program intercepts images of the variation of the water level with time at every instant in the video and analyzes these water level images. The image processing at a certain moment is as follows: first, grayscale processing is performed to obtain a grayscale image (Fig 3A); then, binarization is performed to obtain a binary image (Fig 3B); next, the maximum centroid and the water level line are found; and the water level red line is drawn to obtain a water level red line figure (Fig 3C). Finally, binarization is performed to obtain a water level binary image (Fig 3D), and centroid analysis of this water level line is carried out to obtain the centroid ordinate of the water level line as the accurate water level line. The image processing methods at other times are the same. An H-T diagram is drawn by comparing the water level and time at each moment, that is, the change in the water head difference with time.

## 3. Test results

**Fig 4:**
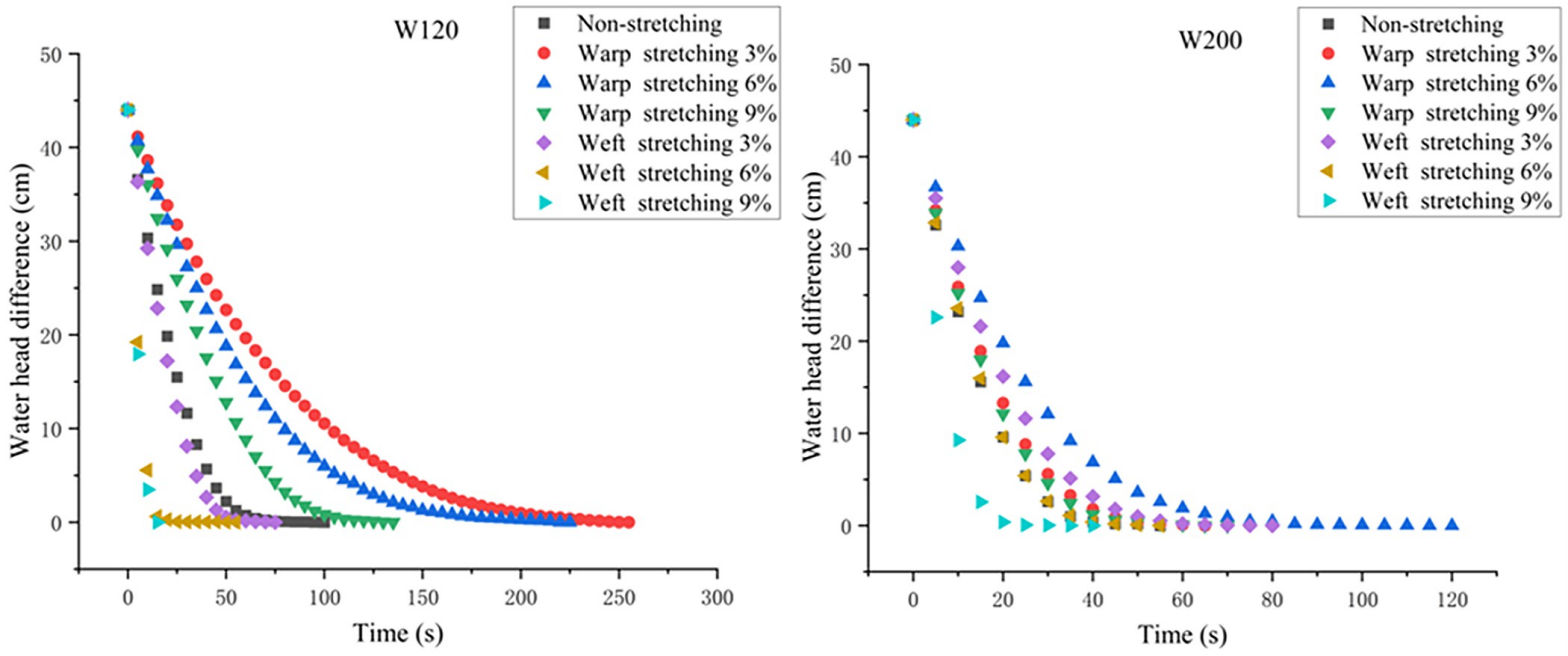
Variation curve of the water head difference with respect to time.

During the test, the video shot by the camera is transmitted to the independently developed program for processing. The water level reading at any time is *h*_*i*_, and the water level reading after the test is *h*_*f*_. The initial water head position *h*_*0*_ is calculated with the formula *H* = 2(*h*_*i*_-*h*_*f*_), the water level difference *h*_*i*_ is then recorded every 5 s, and the curve of the water head difference corresponding to different working conditions with time is created. The initial value is set as *H*_*0*_ = 44 for the convenience of subsequent data processing, as shown in Fig 4. As the seepage time increases, the head difference between the left and right sides of the instrument decreases and eventually reaches zero.

## 4. Analysis of the test results

### 4.1 Laminar calculations

The average flow velocity is used instead of the flow velocity of the entire process. Because of the same diameter of each air cylinder, the water level h of the left air cylinder is equal to the water level h of the right air cylinder during the same test time.

By Darcy’s Law, the following can be used:

v=Kj (1)

where the hydraulic gradient is defined as follows:

$$j=\frac{H}{\delta }$$ (2)

The following is obtained by substituting Formula (2) into Formula (1):

$$v=\frac{k}{\delta }H$$ (3)

Formula (4) is defined as follows:

$$\frac{k}{\delta }=\psi$$ (4)

where ψ is the water permeability.

The following equation can be obtained:

v=ψH (5)

From the integral relations, the following can be used:

$$v=-\frac{dh}{dt}$$ (6)

where dH is defined as follows:

dH=2dh (7)

Formula (7) substituted into Formula (5) produces the following:

$$\psi dt=-\frac{1}{2}\cdot \frac{1}{H}dH$$ (8)

The integration of Formula (8) produces the following:

$$\psi t=-\frac{1}{2}\cdot \ln\;H+C$$ (9)

The integral constant is cleared up, as follows:

$$\psi (t_{2}-t_{1})=\frac{1}{2}\cdot \ln\left(\frac{H_{1}}{H_{2}}\right)$$ (10)

The formula can be simplified, as follows:

$$H=e^{\left(At+B\right)}+C$$ (11)

where *ψ* is defined as follows:

$$\psi =-\frac{A}{2}$$ (12)

**Fig 5:**
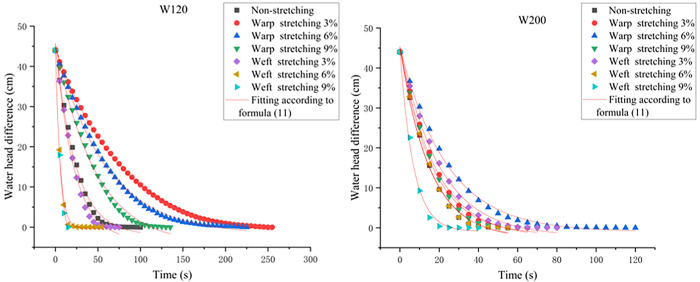
Plots of the water head difference with respect to time by using Formula (11).

The fitting results of each working condition in terms of Formula (11) are shown in Fig 5; here, all the R^2^ values are greater than 0.98. As shown in Fig 5, the fitting curve is the same as the curve obtained from the test, and some fitting values of the curves are slightly different. Therefore, in this study, the water flow in the geotextile is considered to be fluidized turbulent flow.

### 4.2 Turbulence calculation

By Formula (5), the following applies:

$$v^{n}=\Psi H$$ (13)

From the above integral relationship and transformation, the following formula is obtained:

$${\left(-\frac{dH}{2dt}\right)}^{n}=\psi H$$ (14)

From the integral, the following is obtained:

$$H={\left(at+c\right)}^{b}$$ (15)

wherein n and *ψ* are defined as follows:

$$n=\frac{b}{b-1}$$ (16)

$$\psi ={\left(-2ab\right)}^{n}$$ (17)

**Fig 6:**
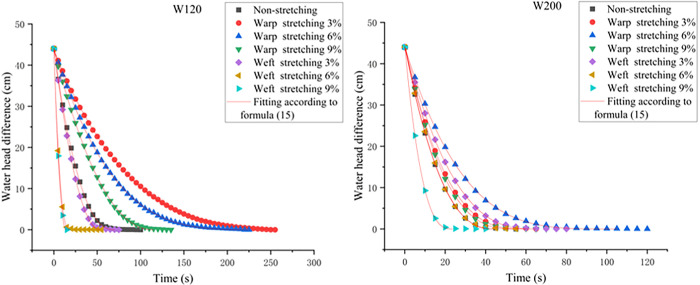
Plots of the water head difference with respect to time using Formula (15).

The fitting results of each working condition according to Formula (15) are shown in Fig 6, and the fitting curve of Fig 6 is closer to the test data compared to that of Fig 5. The R^2^ values are all greater than 0.999. Therefore, Formula (15) was used to calculate the subsequent data processing.

**Fig 7:**
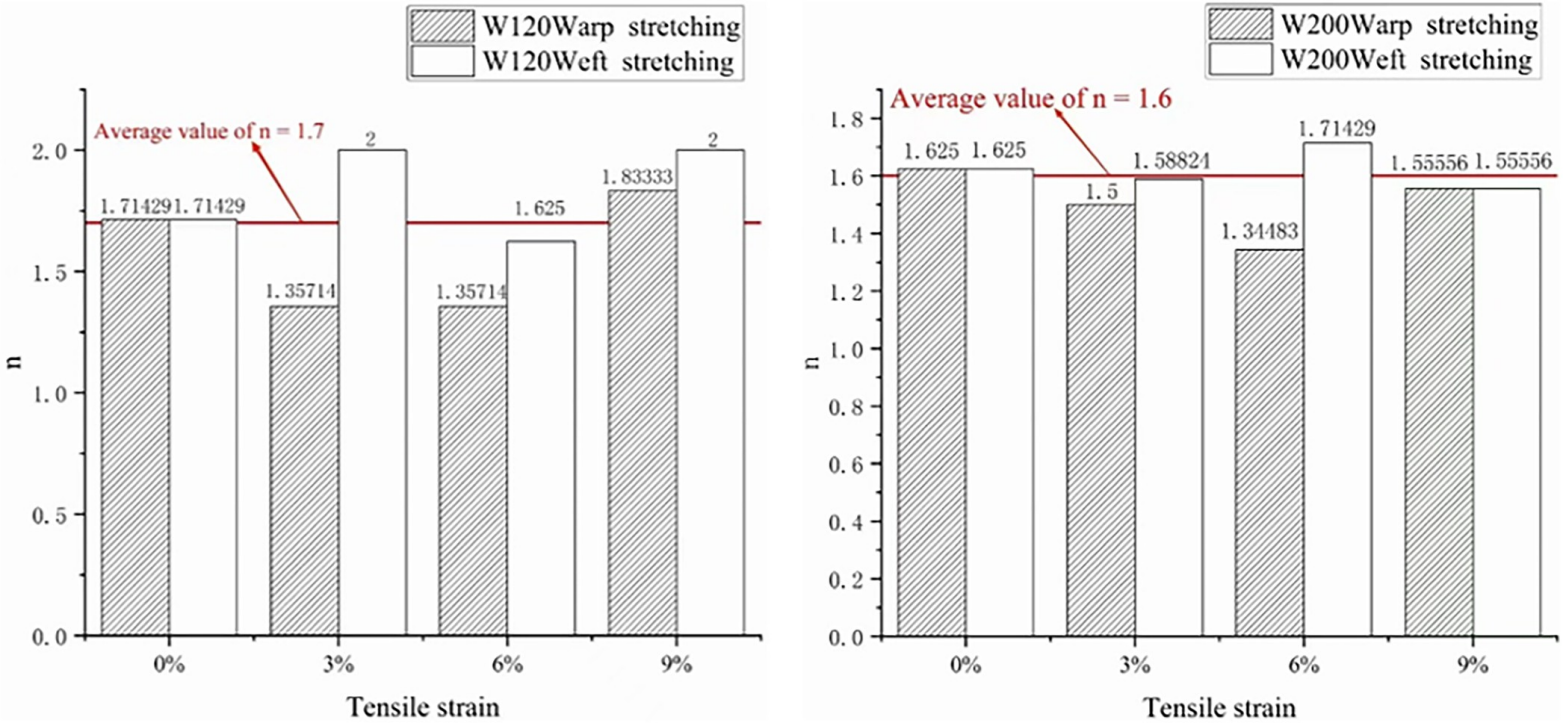
Value n fitting chart.

Considering the calculation of the water permeability of the W120 and W200 geotextiles under turbulent flow, the data for each working condition are further fitted and analyzed by Formula (15) to obtain the value of b, and the value of n is obtained according to Formula (16); finally, water permeabilities of W120 and W200 geotextiles are obtained. The obtained values of n are shown in Fig 7. After calculation, the average values of n under each working condition are 1.7 and 1.6.

Value n of 1.6 and 1.7 are used to fit the data of each working condition another time, and the fitting formula is as follows:

$$H={\left(at+c\right)}^{1.6}\;or\;H={\left(at+c\right)}^{1.7}$$ (18)

**Fig 8:**
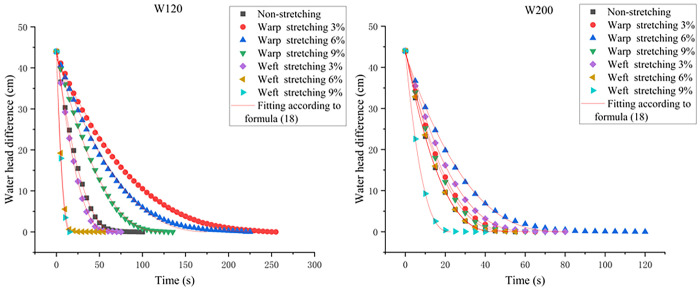
Plot of the water head difference with respect to time using Formula (18).

To facilitate the actual calculations of the subsequent projects, the fitting results of the H-T scatter plot under various working conditions according to Formula (18) are shown in Fig 8, and R^2^ is greater than 0.99.

### 4.3 Influence of uniaxial tension on the water permeability of the geotextile

**Fig 9:**
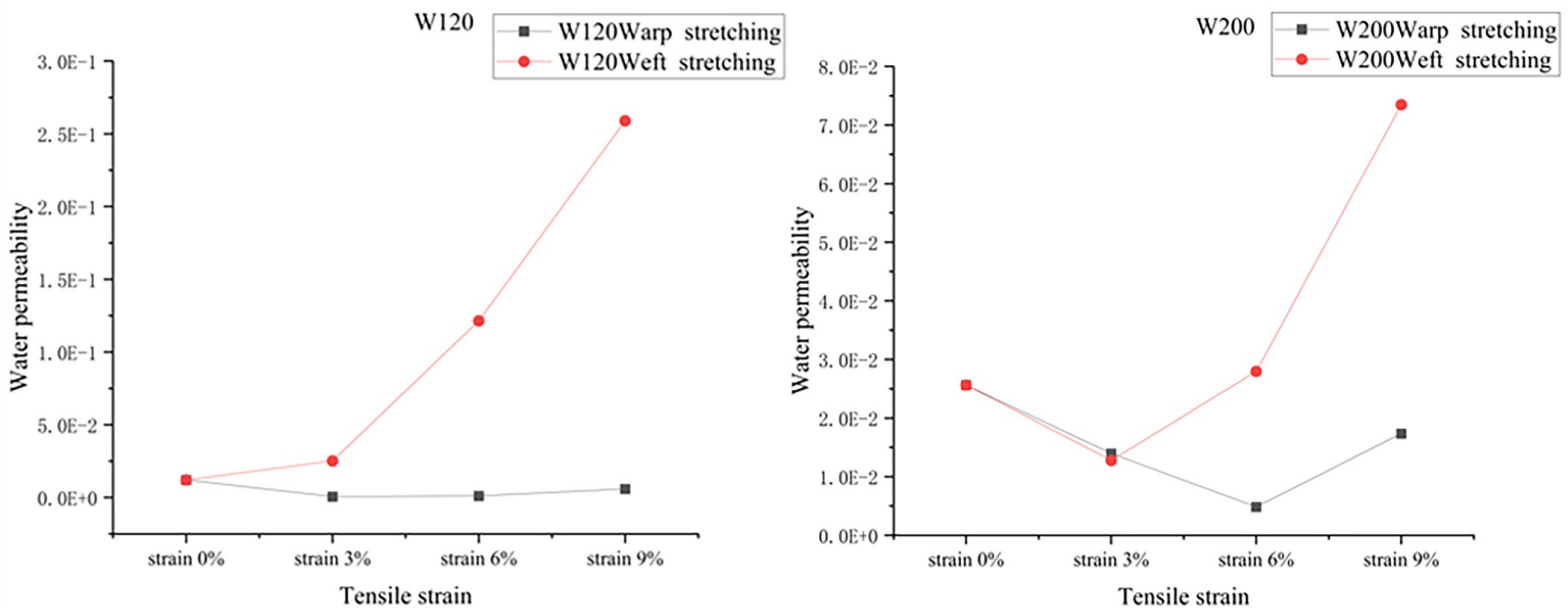
Variation curve of the water permeability of the geotextiles with strain under warp stretching.

By processing the test data, the curve of the water permeability of the geotextile under uniaxial tension is obtained, as shown in Fig 9. Fig 9 shows that the water permeability of the W120 geotextile sample initially decreases and then increases under warp stretching and gradually increases under weft stretching. However, the water permeability of the W200 geotextile samples initially decreased and then increased under both warp and weft stretching. Compared with that of the W200 geotextile sample, the water permeability of the W120 geotextile sample shows greater changes.

**Fig 10:**
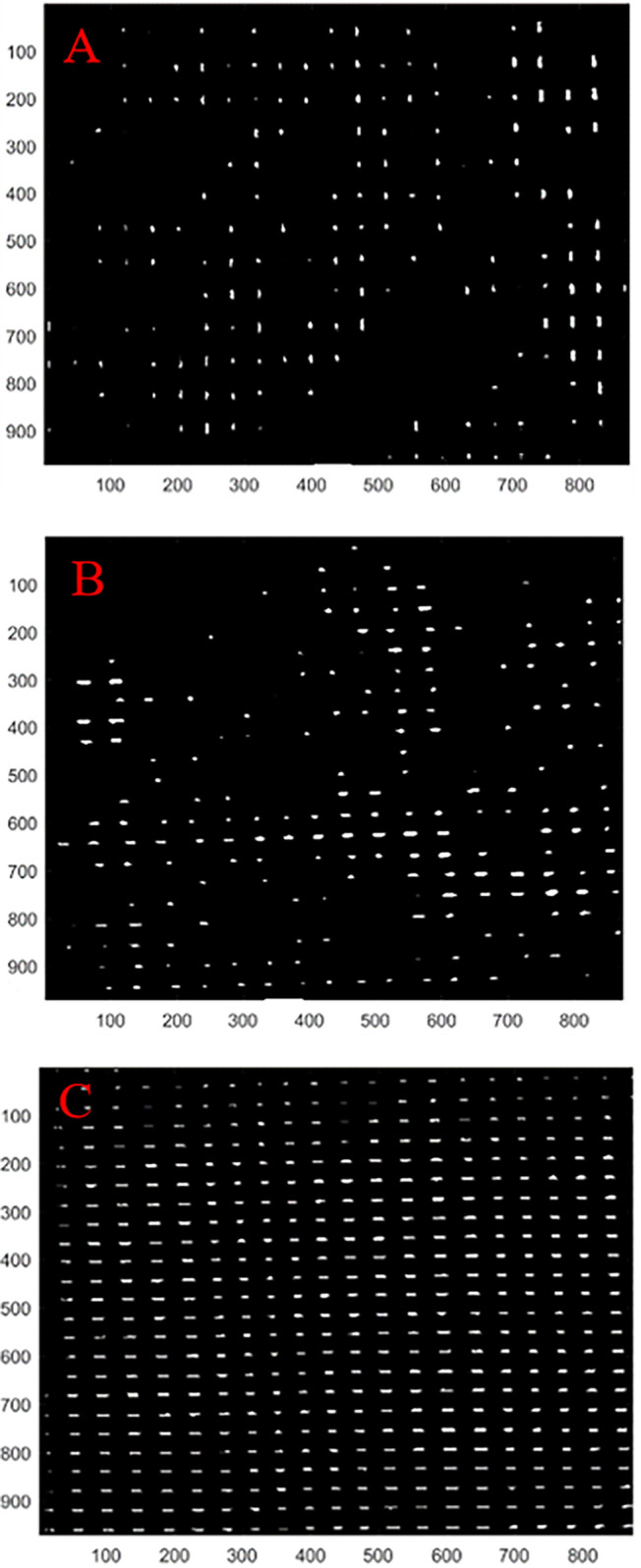
Geotextile samples under different tensile conditions. (A) Geotextile specimen after stretching in the longitudinal direction, (B) Geotextile specimen in the natural state, (C) Geotextile specimen after stretching in the weft direction.

To further reveal the reasons for the influence of uniaxial stretching on the permeability performance of geotextiles, Matlab software was used to process pictures of geotextiles in different states and determine the overall porosity of geotextiles in different states; the results of the processing are shown in Fig 10A, 10B, and 10C represent the geotextile specimens in the warp direction stretching state, the geotextile specimens in the natural state and the geotextile specimens in the weft direction stretching state, respectively.

With an image resolution of 2500×2500 and width and height of 2500 pixels, the porosity of the geotextile specimen in the natural state was calculated to be 1.457% using Matlab. From Fig 10, the warp direction stretching caused part of the fabric filament stripes to undergo certain displacement, the large pore porosity decreased, the compact area was divided into smaller pores, and the overall porosity decreased. The weft direction stretching caused the filament stripes in the overall geotextile specimen to undergo regular displacement; all compact areas of the geotextile under the natural state were dispersed, and the large area produced new pores, which were arranged in a neat and uniform arrangement; moreover, the overall porosity was 1.457% under the natural state. The pores were neatly and uniformly arranged, and the overall porosity increased. The porosity of the geotextile specimen after warp direction stretching was calculated to be 1.061%, and the porosity of the geotextile specimen after weft direction stretching was 3.561%. The fundamental reason for the change in the permeability performance of the geotextile after uniaxial stretching was that uniaxial stretching changed the porosity of the geotextile.

## 5. Conclusion

1. By fitting and analyzing the water head difference and time image of the geotextiles under laminar flow and turbulent flow, the fitting curve was more accurate under turbulent flow; specifically, the water flow in the geotextile was closer to turbulent flow.
2. In the same geotextile strain interval, compared with that of the W200 geotextile sample, the water permeability of the W120 geotextile sample showed greater changes. The water permeability of the W120 geotextile samples varied from 0 to 0.3, while that of the W200 geotextile samples varied from 0 to 0.08.
3. In the case of uniaxial tension, the water permeability of the geotextile changed accordingly. The water permeability of the W120 geotextile samples initially decreased and then increased under warp stretching and gradually increased under weft stretching. However, the water permeability of the W200 geotextile samples initially decreased and then increased under both warp and weft stretching.
4. In this study, the same geotextiles were used with two different specifications; future permeability tests should include a variety geotextiles with different specifications, and further quantitative analysis of the geotextile specifications with respect to its permeability performance need to be performed.

## Supporting information

S1 Data (XLSX)
